# Evaluation of Ozone Removal by Spent Coffee Grounds

**DOI:** 10.1038/s41598-019-56668-5

**Published:** 2020-01-10

**Authors:** Pei-Fu Hsieh, Tsrong-Yi Wen

**Affiliations:** 1 Department of Mechanical Engineering, National Taiwan University of Science and Technology, No 43, Sec 4, Keelung Rd, Taipei, 106 Taiwan; 2 High Speed 3D Printing Research Center, National Taiwan University of Science and Technology, No 43, Sec 4, Keelung Rd, Taipei, 106 Taiwan

**Keywords:** Environmental sciences, Natural hazards, Natural hazards, Environmental sciences

## Abstract

Activated carbon is the most known material used to adsorb ozone. Activating carbonaceous materials by ozonation is commonly used to produce activated carbon, however, requiring sophisticated skills and professional equipment. This paper presents a reversed idea: to adsorb ozone using an unactivated carbonaceous material, coffee. Three powder adsorbents are presented: fresh coffee (unactivated), spent coffee grounds (unactivated), and activated carbon (commercially available). The test is conducted by measuring and comparing the ozone concentration in an ozone-supplied chamber with or without the ozone adsorbent. The results show that, at the specific conditions, the peak ozone concentration is lowered by 38% to 56% when the chamber has the activated carbon. At the same conditions, the peak ozone concentration is lowered by 25% to 43% when the chamber has the coffee powders (either fresh or spent). The elemental analysis demonstrates that the oxygen content after the ozone adsorption increases by 20%, 14.4%, and 34.5% for the fresh coffee, the spent coffee grounds, and the activated carbon, respectively. The characteristic analysis (the Fourier-transform infrared spectroscopy, the thermogravimetric, and the Brunauer-Emmett-Teller) suggests that the unactivated coffee is not porous, however, contains various organic compounds that could react with and consume ozone.

## Introduction

Ozone (O_3_) is smell-irritating, colorless, capable of oxidizing organic compounds, and one of the six criteria air pollutants set by the United States Environmental Protection Agency. Besides, ozone is able to react with terpenoids to form secondary organic aerosols^1,2^ that take a significant mass fraction of aerosol particles^3^. Long-term exposure to an unconditioned ozone environment can cause health problems such as asthma, allergic, airway issues, and even mortality^4–6^. To maintain air quality and to protect people, several countries have set the regulations of the maximum ozone concentration^7,8^.

Chemically, ozone is formed when an oxygen atom (O) attaches to an oxygen molecule (O_2_). However, ozone is unstable and the depletion of ozone is spontaneous in nature. Simply, the formation and the depletion of ozone are shown in Eq. (1) to Eq. (3).

1 $${\rm{e}}+{{\rm{O}}}_{2}\to 2{\rm{O}}\,\cdot +{\rm{e}}$$

2 $${\rm{O}}\,\cdot +{{\rm{O}}}_{2}\to {{\rm{O}}}_{3}$$

3 $${\rm{O}}\,\cdot +{{\rm{O}}}_{3}\to 2{{\rm{O}}}_{2}$$

Because of many known adverse health effects, controlling ozone concentration is required for certain environments such as aircraft cabins^9^ and offices/buildings that come with discharge-based devices^10^, e.g., laser printers^11^ and electrostatic precipitators^12^. Activated carbon is one of the commonly used ozone adsorbents because of its large surface area and specific chemical properties. Mueller *et al*. showed that applying the activated carbon filter in an 11.9 m^3^ aluminum room where has a working electric air cleaner lowers the ozone concentration by 90% in seven minutes^13^. Yang *et al*. presented a novel technique that combines the carbon nanotubes and the activated carbon filter to remove ozone and reported a removal efficiency of 99%^14^.

Activated carbon can be produced by chemically or physically activating carbonaceous materials such as coal, peat, rice husk, wood, and peach stone^15–17^. Coffee, one of the most common daily drinks in modern society, is essentially carbonaceous as well. A few researchers have successfully turned the coffee into the activated carbon. Boonamnuayvitaya *et al*. made the activated carbon from the coffee residues with the different activating agents and reported that the maximum specific surface area was about 914 m^2^ g^−1^^18^. Kante *et al*. made the activated carbon from the spent coffee waste with an agent of zinc chloride under several treatment conditions, and the maximum specific surface area presented was up to 1,121 m^2^ g^−1^^19^. Namane *et al*. demonstrated that the coffee-grounds made activated carbon has a promising removal efficiency for the phenol (>80%) and the dye (>90%) when compared to the commercial activated carbon^20^.

It is certain that activated carbon is capable of adsorbing ozone and can be made from spent coffee grounds by proper activation processes that, however, require sophisticated skills and professional equipment. This paper presents an idea to adsorb ozone by using two types of unactivated coffee powder: fresh coffee and spent coffee grounds. The commercially available activated carbon powder is also introduced for the comparison purpose. This paper presents the ozone concentration and ozone removal efficiency when there present the mentioned adsorbents in a chamber. Four characteristic analysis are then brought out to see how the coffee powders react with ozone, including the energy-dispersive X-ray spectroscopy (EDS), the Fourier-transform infrared spectroscopy (FTIR), the thermogravimetric analysis (TGA), and the specific surface area (Brunauer-Emmett-Teller, BET).

## Material and Methods

The experimental setup includes three components connected in series. The ozone generator provides the ozone flow to a chamber that connects to an ozone analyzer (Ecotech Serinus 10) used to measure the ozone concentration. The ozone adsorbent under test is placed in the chamber to react with ozone.

### Ozone generator

A dielectric barrier discharge based ozone generator is used to provide a stable ozone flow^21,22^. The ozone generator consists of an aluminum cylinder (electrode) and a quartz tube (dielectric material). A pump is used to supply air to the ozone generator at a constant rate of 4 × 10^−5^ m^3^ s^−1^ (2.4 liters per minute).

### Chamber

**Figure 1:**
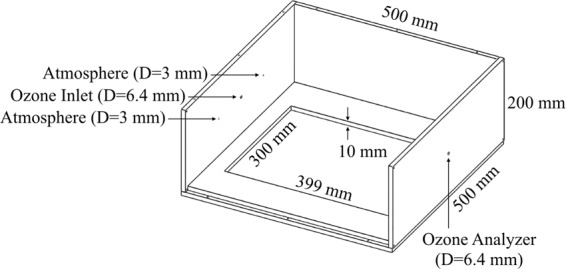
The schematic and the dimension of the chamber.

The chamber is made of 10-mm-thick transparent acrylic plates, as shown in Fig. 1. The exterior dimension of the chamber is 500 mm by 500 mm by 200 mm. There are four openings on the walls of the chamber. One is the ozone inlet (at the center of the wall), the one right opposite to the ozone inlet connects to the ozone analyzer, while the remaining two are open to the atmosphere to balance the flow and the pressure. The ozone adsorbent is placed in a shallow basin in the bottom center of the chamber.

### Ozone adsorbent

#### Preparation

This paper tests three powder adsorbents: fresh coffee, spent coffee grounds, and activated carbon. Table 1 summarizes the information and the conditions of these adsorbents. Note that all the coffee powders used in this paper are unactivated.

**Table 1:** The information and the conditions of the three ozone adsorbents.

Adsorbent	Physical Format	Amount	Water Content
Fresh Coffee	Powder	100 g	1.7% ± 0.3%
(Starbucks, Breakfast Blend Medium Roast)		300 g	
Spent Coffee Grounds	Powder (Brewed then baked at 100°C for 6 hours)	100 g	96.2% ± 5.2%
(Starbucks, Breakfast Blend Medium Roast)		300 g	
Activated Carbon	Powder	100 g	7.0% ± 0.6%
(The First Chemical Works, 3 × 4 Mesh)		300 g	

All these powder adsorbents are prepared by grinding their fresh beans using the same grinder (Cuisinart DBM-8TW) at the same size setting (Medium). The spent coffee grounds are the wastes of brewing the fresh coffee powder by a regular coffee machine (Frigidaire FKC1151HS). Before getting tested, the spent coffee grounds are baked in an oven at 100°C for 6 hours.

To understand if the amount of the ozone adsorbent would affect the performance of the ozone removal, this paper tests the adsorbent at two weights. When the amount of the ozone adsorbent is 100 grams, one-third of the shallow basin (close to the ozone inlet) is evenly filled with the ozone adsorbent, while the remaining two-thirds is placed with the acrylic plates. When the amount of the ozone adsorbent is 300 grams, the entire shallow basin is evenly filled with the ozone adsorbent. Note that the entire shallow basin is placed with the acrylic plates when conducting the test that is without any ozone adsorbent.

#### Physical inspections

**Figure 2:**
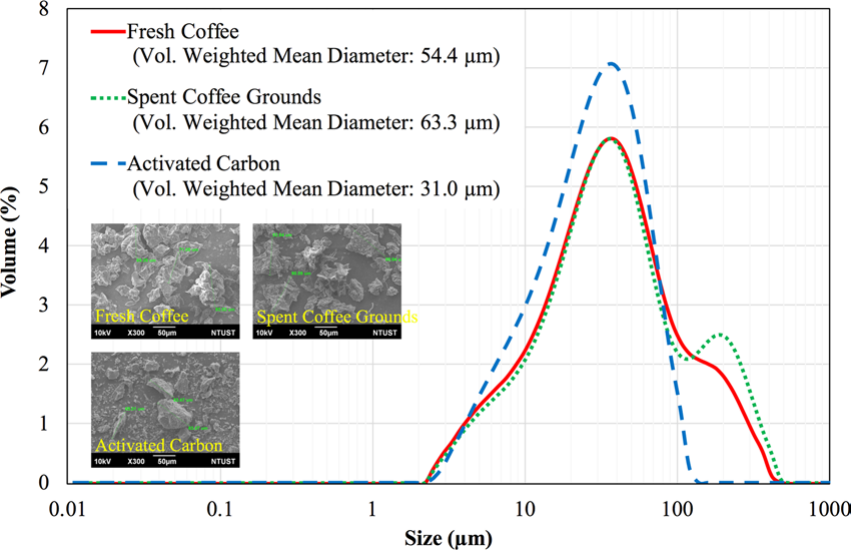
The particle size distribution of the three ozone adsorbents.

Figure 2 shows the particle size distribution in terms of equivalent sphere diameters using a particle size analyzer (Malvern Mastersizer 2000) and the shape inspection using a scanning electron microscope (SEM, JEOL JSM-6390LV).

Although the particle size distribution of these three ozone adsorbents is similar, the fresh coffee and the spent coffee grounds have some large debris. The spent coffee grounds have the largest volume-weighted mean diameter probably because some of the debris agglomerates to the bigger ones. On the other hand, the activated carbon bean has a higher Young’s Modulus and is thus more brittle when compared with the coffee bean^23^, making the activated carbon powder finer. Moreover, the SEM images show that the shape of all the ozone adsorbents is irregular, as expected.

### Procedures for measuring ozone concentration

The ozone analyzer measures and records the ozone concentration of the chamber every 10 seconds and nonstop for 23 minutes, while the ozone generator turns on only for the first three minutes and then turns off. Note that although the ozone generator shuts down exactly at the third minute, the ozone concentration of the chamber read by the ozone analyzer still increases for another half a minute. This is because the chamber is big and the ozone analyzer needs some time to read the ozone concentration that represents the one after the ozone generator is turned off. In order to ensure the repeatability, five measurements are conducted for every testing leg.

### Procedures for conducting characteristic analysis

This paper inspects the ozone adsorbent by the FTIR, the TGA, and the BET to identify the changes before and after the ozone adsorption.

For the FTIR analysis, the sample (ozone adsorbent) of one milligram is first dried out completely, and then, is mixed with 100 mg potassium bromide (KBr) to make a pellet. The pellet is compressed by a heavy hydraulic jack (12 Ton E-Z Press^TM^) at 10 ton cm^−2^ to make a thin film. The thin film sample is then analyzed immediately using the FTIR spectroscopy (Bio-Rad FTS-3500) to avoid possible humidity issues. The background spectrum is obtained using a pure KBr pellet by the same mass and the same manner mentioned above. The FTIR results are presented by the spectrums that are subtracted the background ones from the sample ones to highlight the changes.

The TGA analysis is carried out by the thermogravimetric analyzer (TA Instruments TGA Q500). One gram sample is heated in the temperature range of 0°C to 800°C at a rate of 20 °C min^−1^ under an airflow.

Before getting tested in the BET analyzer (MicrotracBEL BELSORP-max) with nitrogen, the sample is heated up to 150°C under a vacuum condition (<10^−2^ kPa) for four hours (MicrotracBEL BELPREP-vac II) in order to be completely dehydrated. The nitrogen adsorption-desorption isotherms are logged at 77 K. The specific surface area is calculated by the BET method, while the total pore volume is got based on the amount of the nitrogen adsorbed at a 0.99 relative pressure.

## Results and Discussion

### Ozone concentration

**Figure 3:**
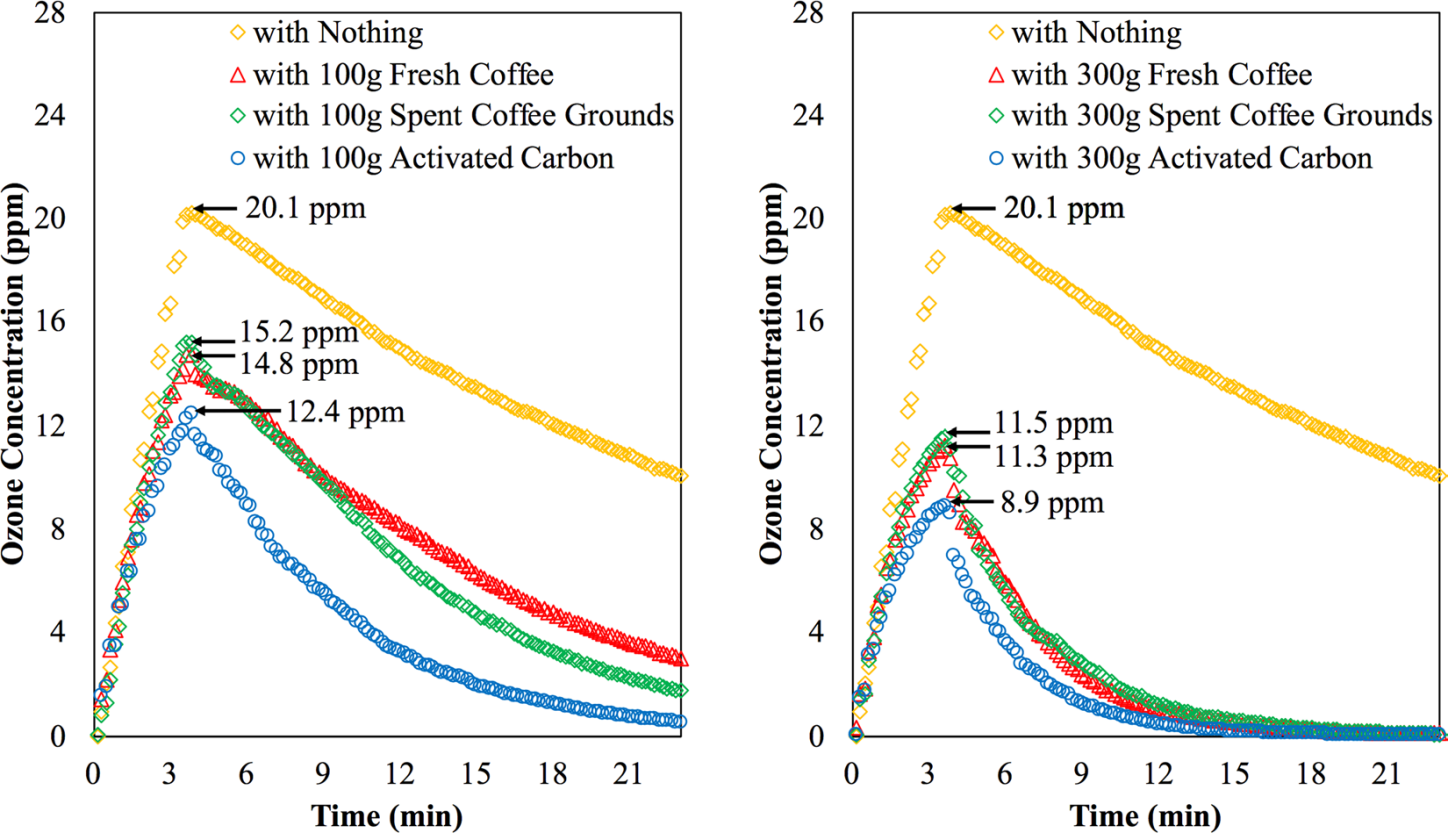
The ozone concentrations over time with and without the ozone adsorbent. The peak ozone concentrations are marked accordingly.

Figure 3 shows the ozone concentrations of the chamber over time, while the peak ozone concentrations are numerically labeled.

During the ozone generation period (before the 3^rd^ minute), when the chamber comes with the ozone adsorbents, the rate of the ozone generation is not as linear as the case that comes without the ozone adsorbents, and the ozone concentration is thus suppressed. It is also apparent that the more the ozone adsorbents are presented in the chamber, the stronger the effects of the ozone suppression are. This is because when the chamber has more adsorbents, there are more surface areas that can react with ozone.

Although the fresh coffee, the spent coffee grounds, and the activated carbon do react with ozone, the capabilities of the ozone suppression of the fresh coffee and the spent coffee grounds look similar while the activated carbon works best as anticipated. The peak ozone concentration (at the 3.5^th^ minute) is lowered by 26.4%, 24.4%, and 38.3% when the chamber presents 100 grams of the fresh coffee, the coffee ground, and the activated carbon, respectively. Furthermore, the peak ozone concentration is lowered by 43.8%, 42.3%, and 55.7% when the chamber presents 300 grams of the fresh coffee, the coffee ground, and the activated carbon, respectively.

### Ozone removal efficiency

**Figure 4:**
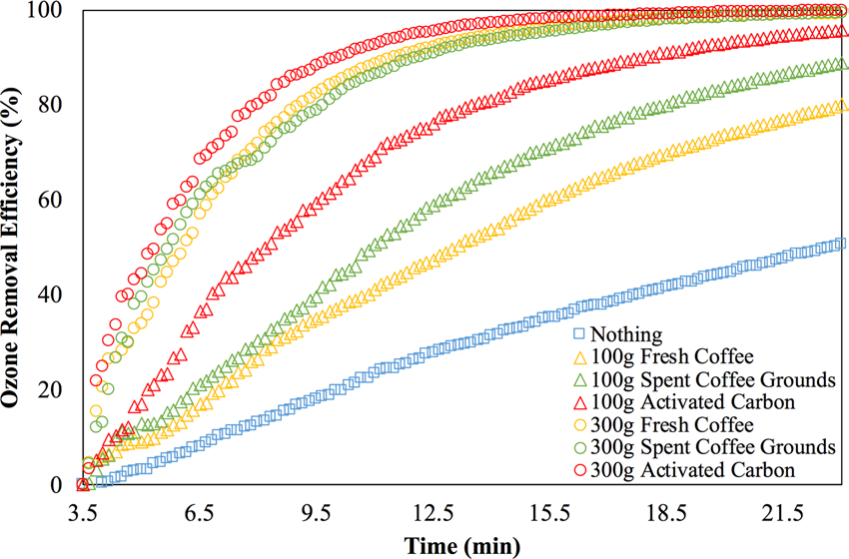
The ozone removal efficiency over time with and without the ozone adsorbent.

Figure 4 shows the time-dependent ozone removal efficiency *η*_removal_ defined by Eq. (4), for the ozone depletion period (after the 3.5^th^ minute).

4 $${\eta }_{{\rm{removal}}}=1-\frac{{C}_{{\rm{t}}}(t)}{{C}_{{\rm{i}}}}$$

where *C*_i_ is the initial ozone concentration and *C*_t_(*t*) is the ozone concentration with respect to time.

All the ozone adsorbents do speed up the ozone removal, but the rate of the ozone removal declines and saturates over time. This is because the ozone desorption phase in a chamber theoretically decays over time in an exponential manner^24^ and the adsorption capability of a substance has a cap^25^.

On the other hand, Fig. 4 also shows that the spent coffee grounds work as good as the fresh coffee, even better when the amount is low. This is because ozone is water-soluble, while the spent coffee grounds used in this case still contains some water. Such the results give one more reason to recycle the spent coffee grounds for ozone removal. Note that the mean diameters of these three adsorbents are different such that the results shown in Fig. 4 may favor the activated carbon because of its small mean diameter. In other words, if the mean diameter of the spent coffee grounds could be as small as that of the fresh coffee or even as that of the activated carbon, the capability of the ozone removal of the spent coffee grounds could be higher.

### Characteristic analysis

#### Elemental analysis

Table 2 shows the elemental analysis by an EDS (JEOL JSM-6390LV). The majority of the elements of all the ozone adsorbents is carbon, and the percent weight of carbon does not change too much after adsorbing ozone (from 1.1% to 2.1%). The increasing of the oxygen after the ozone adsorption gives a clue that these three adsorbents do react with ozone, while the activated carbon changes the most and the spent coffee grounds change the least.

**Table 2:** The EDS results for the three ozone adsorbents before and after the ozone adsorption.

Adsorbent	Ozone Adsorption	Carbon (wt. %)	Oxygen (wt. %)
Fresh Coffee	Before	52.1	16.0
	After	53.1	19.2
	Percent Change	+1.9%	+20%
Spent Coffee Grounds	Before	62.1	11.8
	After	63.4	13.5
	Percent Change	+2.1%	+14.4%
Activated Carbon	Before	83.8	2.9
	After	84.7	3.9
	Percent Change	+1.1%	+34.5%

The percent change is calculated by (X_after_ − X_before_)/X_before_ × 100%, where X represents the percent weight of carbon or oxygen.

#### FTIR analysis

**Figure 5:**
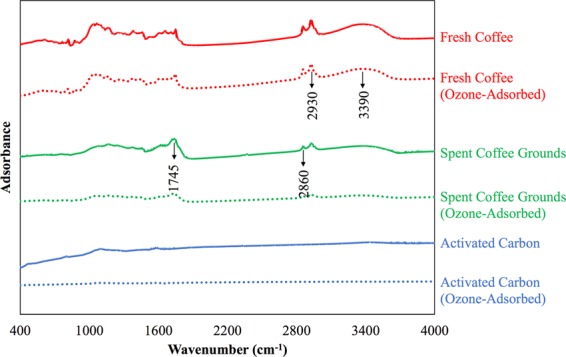
The FTIR results for the three ozone adsorbents (arbitrary adsorbance-axis).

Figure 5 shows the analysis of all the ozone adsorbents before and after the ozone adsorption using the FTIR spectroscopy to check if there are any changes in the functional groups.

The fresh coffee and the spent coffee grounds have nearly identical spectrums before and after the ozone adsorption. In other words, the functional groups of both the coffees are not chemically modified much after reacting with ozone for 23 minutes. On the contrary, the activated carbon has dramatically different spectrums before and after the ozone adsorption. This implies that the functional groups of the activated carbon have chemically changed after the ozone adsorption, coincidence with those reported in the literature^25–27^.

Furthermore, the most significant difference in the band between the coffees (both fresh coffee and spent coffee grounds) and the activated carbon is that the coffees have three major peaks (1745 cm^−1^, 2860 cm^−1^, and 2930 cm^−1^) and one minor peak (3390 cm^−1^) while the activated carbon does not. The types of vibrations responsible for the peaks mentioned above are C=O stretching, C–H stretching (aliphatic), and O–H/N–H stretching^28^. Such a difference indicates that the coffee comprises of many organic compounds that are highly oxidative^29^. This is believed to be one of the reasons that coffee helps consume ozone.

#### TGA analysis

**Figure 6:**
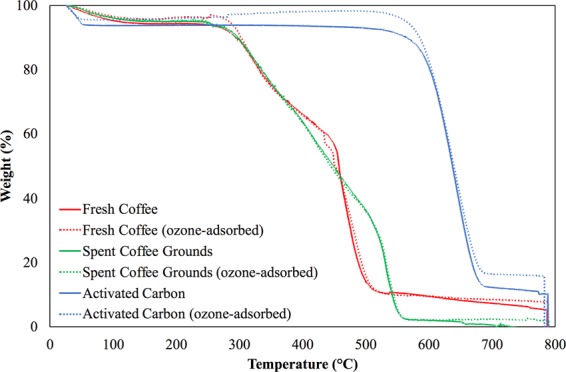
The TGA results for the three ozone adsorbents.

The TGA analysis is to examine the pyrolytic behaviors of the ozone adsorbents. In general, there are three thermal stages for organic substances. The first stage is water removal. The second stage could consist of several sub-stages that associate with different chemical decompositions. The last stage is the complicated changes in the structure of the lignin. Figure 6 shows the TGA results for all the three ozone adsorbents.

It is not surprising that the results of the activated carbon agree well with those reported in the literature^30,31^. The following observations and the explanations are made to both the fresh coffee and the spent coffee grounds because they have similar behaviors. The water removal stage happens before ~200°C, during which ~8% of the weight is removed in this stage. The following two sub-stages that reduce 70% to 90% of the weight could be the thermal depolymerization of hemicelluloses and cellulose degradations^32^. In other words, these two sub-stages are the carbonization stage^33^, suggesting that the coffee is an organic compound rich substance. The last stage starts around ~500°C to ~550°C, turning the remaining mass into ashes.

#### BET analysis

This paper also evaluates the specific surface area and the total pore volume of all the testing ozone adsorbents using a BET analyzer, while the results are shown in Table 3. For the activated carbon after the ozone adsorption, the specific surface area and the total pore volume decrease, similar to the results reported in the literature^34^. For the unactivated coffee, both fresh and spent, the specific surface area is three orders smaller and the total pore volume is four orders smaller than those of the activated carbon. Therefore, unactivated coffee can be considered non-porous.

**Table 3:** The results of the BET analysis.

Adsorbent	Ozone Adsorption	S_BET_ (m^2^ g^−1^)	V_T_ (cm^3^ g^−1^)
Fresh Coffee	Before	0.349	0.080
	After	0.081	0.019
	Percent Change	−76.8%	−76.3%
Spent Coffee Grounds	Before	0.305	0.070
	After	0.185	0.043
	Percent Change	−39.3%	−38.6%
Activated Carbon	Before	981	225
	After	964	222
	Percent Change	−1.7%	−1.3%

S_BET_ is the specific surface area and V_T_ is the total pore volume. The percent change is calculated by (X_after_ − X_before_)/X_before_ × 100%, where X represents S_BET_ or V_T_.

Before the ozone adsorption, both the specific surface and the total pore volume of the fresh coffee are larger than those of the spent coffee grounds. This is probably because the spent coffee grounds are water-reacted coffee powders such that some compounds might be formed, changing the amount of the surface area and the pore volume when compared with the fresh coffee.

After ozone adsorption, the specific surface area and the total pore volume of both types of coffee decrease, meaning that the pores of these two types of coffee are obstructed, physically or chemically. While the decreasing rates of both the specific surface area and the total pore volume of the fresh coffee are larger than those of the spent coffee grounds, the capability of the ozone adsorption of the spent coffee grounds is as good as that of the fresh coffee. A possible reason causing the above phenomena could also be the water content in the spent coffee grounds. The spent coffee grounds are not dried out before getting tested, while ozone is water-soluble. In other words, although water occupies some pores in the spent coffee grounds, water also helps dissolve ozone, making the spent coffee grounds adsorb ozone as good as the fresh coffee does.

## Conclusions

This paper presents how the unactivated coffee powder helps ozone removal. The results show that having some fresh coffee or spent coffee grounds in an ozone-supplied chamber does suppress the ozone concentration by up to 43%, competitive to the performance of the commercial activated carbon (about 56%). Recycling spent coffee grounds to remove ozone could be further developed for specific purposes.

The characteristic analysis suggests that unactivated coffee is not highly porous but is an organic compound rich material. The FTIR analysis shows that the unactivated coffee contains a certain amount of hydrocarbon bonds that are the characteristics of organic compounds. Besides, the surface functional groups of the unactivated coffee do not change too much before and after the ozone adsorption because the FTIR spectrums do not change significantly. The TGA analysis indicates that the unactivated coffee has a clear carbonization stage, the proof of its unactivated characteristic and thus organic compound rich. On the other hand, the BET analysis demonstrates that the unactivated coffee is not as porous as the activated carbon is. The BET results also show that both the specific surface area and the total pore volume of the unactivated coffee significantly decrease after the ozone adsorption, implying that something created by the reactions between the ozone and the unactivated coffee obstructs the pores of the unactivated coffee.
